# Prognosis in Operation for Appendicitis

**Published:** 1899-06-03

**Authors:** 


### PROGNOSIS IN OPERATION FOR APPENDICITIS.

In almost every case in which it is a question whether or not to undertake operative treatment the answer must depend not only upon the gravity of the condition for which it is proposed, but also upon the severity and the danger of the operation itself. This more especially applies in cases of chronic appendicitis, a condition in which, when suppuration has not yet occurred, the danger lies mostly in the future, and in which, at any rate temporary relief can almost certainly be obtained by very simple measures. For such a disease any operation involving serious risk to life, or even involving material risk of abiding evil consequences, such as hernia, is clearly out of court. Indeed, one may almost say that the propriety of operating in such cases depends almost entirely upon the confidence we feel in being able to do what is necessary without doing any harm. We would therefore refer to a paper by Dr. Edebohls,[1] in which he states that he has operated in such conditions 132 times without losing a patient, that "with up-to-date technics a patient may leave his bed well within a week, and be back at his usual occupation at the end of two weeks or less," and that "with faultless and proper technics hernia and a disfiguring scar can be absolutely guaranteed against, and this irrespective of the length of the incision required in the particular case." We are not very fond of positive statements of this kind, implying as they seem to do a sort of reproach on one's less fortunate brethren when their cases do not do quite so well; but Dr. Edebohls is very confident about his facts. He employs either Battle's incision,[2] or McBurney's gridiron incision,[3] or his own lumbar incision in cases in which right nephropexy is employed at the same time as appendectomy. Whichever incision is employed, the skin is closed by the intracuticular suture to avoid a disfiguring scar, and inversion of the entire uncut appendix is preferred in all cases in which it is practicable. In cases in which suppuration is present the matter is somewhat different, but it is stated that even in such cases, and where extensive drainage is found to be indispensable, "the resources of our art enable us to reduce the period of confinement to two weeks or less on the average, and to guarantee our patients against hernia." The essentials of the operation seem to be the protection of the general peritoneum from infection by means of gauze packing during the operation, if the abscess be not attached to the anterior abdominal wall; the careful mopping out of each abscess cavity as it is opened before proceeding to a fresh one, and its disinfection by peroxide of hydrogen or gauze moistened with sublimate solution; the arrangement of a loose and slender column of gauze for drainage in a straight line from the inverted stump to the opening in the anterior abdominal wall; getting rid of this drainage gradually within about five days; and as soon as the cavity is obliterated refreshing the edges by dissecting off the granulations all around in one clean piece, and closing the wound, the separated muscular fibres and the aponeurosis of the external oblique being brought together "by a continuous suture of forty-day catgut carried in two tiers and tied with a single knot." On the next or the third day the patient bids adieu to his bed. Dr. Edebohls is very confident in the certainty of success, and he says "there seems to be no longer any good reason why all patients suffering from appendicitis, acute or chronic, should not have the benefit of operation." We take it that none of the cases referred to are cases in which diffuse peritonitis has occurred. In these the question of operation has to be discussed on quite another basis.

[1] Med. Rec., May 13. [2] Brit. Med. Jour., 1895, ii., page 1,360. [3] Ann. Surg., 1894, xx., page 38.

